# Tracheal Hemangioma: The “Cherry” in the Trachea

**DOI:** 10.1155/2016/5682904

**Published:** 2016-03-06

**Authors:** Anne Ann Ling Hsu, Angela Maria Pena Takano

**Affiliations:** ^1^Department of Respiratory & Critical Care Medicine, Singapore General Hospital, Singapore; ^2^Department of Pathology, Singapore General Hospital, Singapore

A young adult presented with a four-month long history of hemoptysis. A computed tomography (CT) scan showed a mid-trachea lesion (Figure 1). A flexible bronchoscopy was performed and revealed a cherry-like polyp (Figure 2(a)) and biopsy resulted in brisk bleeding. The polyp was coagulated at the base with a Nd-YAG (neodymium doped-yttrium aluminium garnet) laser followed by forceps resection via the rigid bronchoscope (Video). Histology of the 8 mm by 10 mm lesion was classic of a lobular capillary hemangioma (LCH) (Figure 3). The patient had been asymptomatic since and bronchoscopy performed two years later showed no tumor recurrence (Figure 2(b)).

Lobular capillary hemangioma (LCH) is typically found on cutaneous and oronasal mucosa. Tracheobronchial LCH is a rarity [1]. Hemoptysis often occurs for a short duration ranging from weeks to months with the exception of one reported case of massive hemoptysis which required arterial embolization. The airway lesion is usually small (<10 mm), sessile, or polypoid with a distinctive glinting vascular (cherry) appearance that bleeds easily. Pathogenesis of this benign tumor is unclear and has been correlated to infections, trauma, and hormonal shifts [1]. The latter can be supported by the case of a rapidly growing trachea LCH (40 mm by 20 mm) found in a pregnant lady who presented with critical airway obstruction [2]. The characteristic findings of a homogeneously contrast enhanced lesion seen on CT scan and an airway lesion (as described above) observed during bronchoscopy should lead to a cautious biopsy to clinch the diagnosis.

Bronchoscopic ablative intervention often results in a cure although past reports had a follow-up of one year or less. Ablative modalities included endoscopic resection with Nd-YAG laser, argon plasma coagulation, electrocautery, cryotherapy, and forceps. In one reported case of tumor recurrence, brachytherapy was applied.

## Figures and Tables

**Figure 1:**
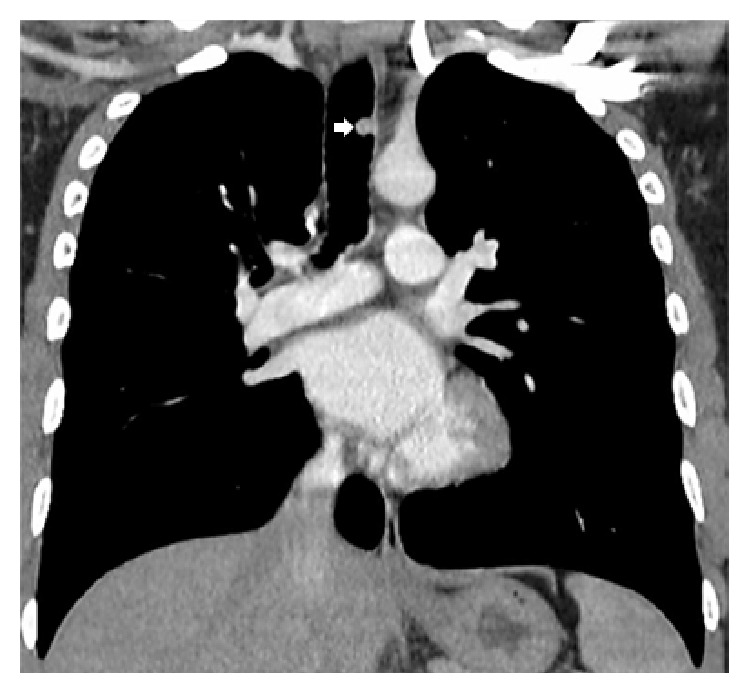
Computed tomography showed a homogeneously contrast enhanced polyp (white arrow) on the left lateral wall of the mid-trachea.

**Figure 2:**
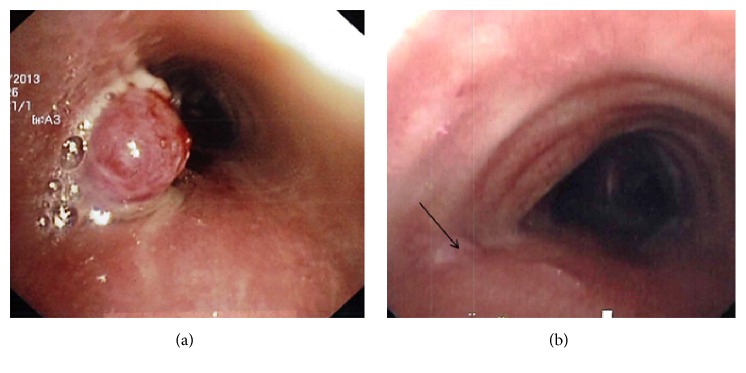
(a) Distinct cherry-like polyp consistent with hemangioma arising from the left lateral wall of the mid-trachea depicted on bronchoscopy. (b) Bronchoscopy performed two years later revealed a small scar induration with normal mucosa (arrow) overlaying it at the base of the previously resected hemangioma.

**Figure 3:**
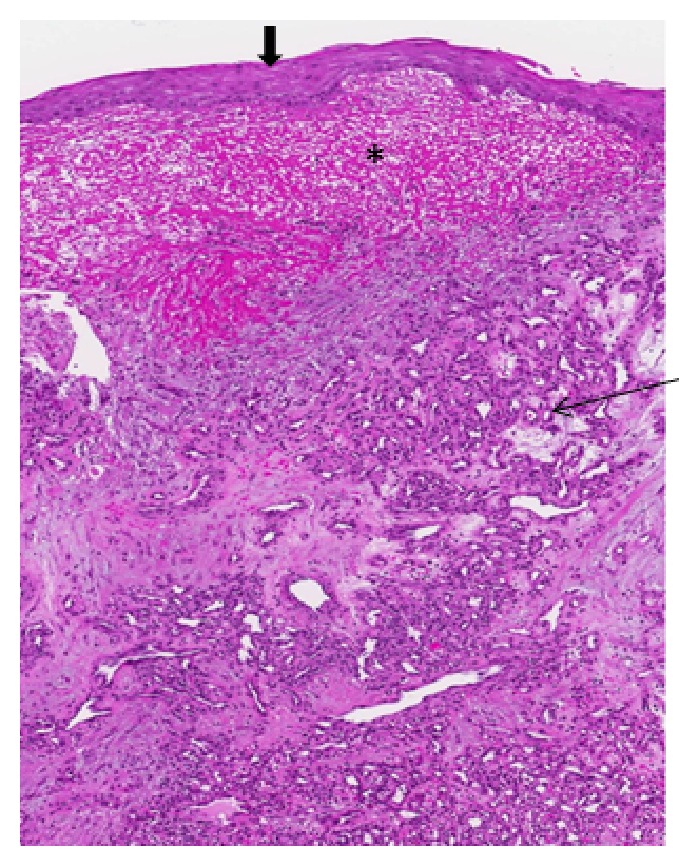
A nodular lesion with squamous metaplasia (marked downwards thick arrow) of the surface epithelium, underlying fibrinous exudates (marked *∗*), and a florid proliferation of small capillaries (marked →) surrounded by fibrous stroma on 10x Hematoxylin and Eosin section.
